# Guiding migration of transplanted glial progenitor cells in the injured spinal cord

**DOI:** 10.1038/srep22576

**Published:** 2016-03-14

**Authors:** Xiao-bing Yuan, Ying Jin, Christopher Haas, Lihua Yao, Kazuo Hayakawa, Yue Wang, Chunlei Wang, Itzhak Fischer

**Affiliations:** 1 Spinal Cord Research Center, Department of Neurobiology and Anatomy, Drexel University College of Medicine, Philadelphia, PA, USA; 2 Hussman Institute for Autism, Baltimore, MD, USA

## Abstract

Transplantation of glial-restricted progenitors (GRPs) is a promising strategy for generating a supportive environment for axon growth in the injured spinal cord. Here we explored the possibility of producing a migratory stream of GRPs via directional cues to create a supportive pathway for axon regeneration. We found that the axon growth inhibitor chondroitin sulfate proteoglycan (CSPG) strongly inhibited the adhesion and migration of GRPs, an effect that could be modulated by the adhesion molecule laminin. Digesting glycosaminoglycan side chains of CSPG with chondroitinase improved GRP migration on stripes of CSPG printed on cover glass, although GRPs were still responsive to the remaining repulsive signals of CSPG. Of all factors tested, the basic fibroblast growth factor (bFGF) had the most significant effect in promoting the migration of cultured GRPs. When GRPs were transplanted into either normal spinal cord of adult rats or the injury site in a dorsal column hemisection model of spinal cord injury, a population of transplanted cells migrated toward the region that was injected with the lentivirus expressing chondroitinase or bFGF. These findings suggest that removing CSPG-mediated inhibition, in combination with guidance by attractive factors, can be a promising strategy to produce a migratory stream of supportive GRPs.

A central challenge following spinal cord injury (SCI) is to promote the growth of injured axons in order to reestablish synaptic connections and functional recovery. Previous studies have shown that despite the poor regenerative capacity of central nervous system (CNS) neurons, they can be encouraged to grow into grafts of peripheral nerve^1^,^2^,^3^. However, axons stop at the boundary of the graft and the host tissue^1^,^2^,^3^,^4^, underscoring the influence of the inhibitory environment of an injured CNS. One promising strategy to support the growth of CNS axons is the transplantation of cells or tissues that can modify the local host environment and support the growth of regenerating axons^5^, including Schwann cell (SCs)^6^,^7^,^8^, olfactory ensheathing cells (OECs)^9^,^10^,^11^, bone marrow mesenchymal stromal cells (MSCs)^12^, neural stem cells (NSC)^13^,^14^,^15^, and glial-restricted progenitors (GRPs)^16^,^17^,^18^. These transplants generate a permissive environment for axon growth, likely by secreting growth factors and forming adhesive extracellular matrix to overcome the inhibitory environment of the injury^18^,^19^. However, similar to grafts of peripheral nerve, the value of transplanting these cells to promote axon regeneration is limited by the fact that most regenerating axons remain within the graft after their initial invasion, failing to grow out of the graft^20^,^21^,^22^,^23^. Therefore, a practical challenge for therapeutic cell transplantation in SCI, in the context of long distance regeneration and connectivity, is to develop strategies to promote axonal growth beyond the graft into putative target areas and to further facilitate synaptic connectivity.

It has been recognized that the migratory properties of grafted cells are beneficial for axon regeneration and functional recovery^24^,^25^. The migration of axon growth-supporting cells out of the graft site may allow for the further modification of the host environment outside of the immediate injury site along the path of nerve regeneration. As an example, the distance of axonal regeneration is closely related to the migration rate of grafted OECs^26^,^27^. Considering the close association between regenerating axons and grafted cells, migration of these cells may even enhance axon growth by a towing mechanism^28^,^29^,^30^.

In this study, we examined molecules that may either restrict or promote the migration of GRPs derived from embryonic spinal cord, a promising cell type to support axon regeneration upon transplantation into sites of SCI^17^,^18^,^21^, and explored methods of inducing the directional migration of grafted GRPs in a dorsal column hemisection (DCH) model of SCI by locally manipulating the expression of these factors. We found that CSPG, a classical axon growth inhibitor, strongly inhibits the adhesion and migration of GRPs, and identified the growth factor bFGF as an attractive factor to promote GRP migration. Directional migration of grafted GRPs *in vivo* can be achieved by manipulating the expression of either chondroitinase (Chase) or bFGF using lentivirus vectors. The methods described here will lay a framework for the future exploration of promoting axon regeneration by guiding the directional migration of grafted GRPs and highlight the importance of developing new strategies to remove or circumvent the inhibitory effects of CSPG.

## Materials and Methods

### Ethics statement

All experimental methods relevant to the use of animals were performed in accordance with the approved guidelines and have been approved by the Institutional Animal Care & Use Committee of Drexel University College of Medicine (protocol NO. 20222).

### GRP culture

GRPs were cultured as described previously^20^,^21^. Briefly, spinal cord tissues of alkaline phosphatase (AP) transgenic rats at embryonic day (E13.5–14) were dissected and dissociated to prepare neural progenitor cells (NPC). NPCs were cultured for 10 days on poly-l-lysine (PLL, 15 μg/mL)/laminin (15 μg/mL) -coated culture dishes in GRP basal medium (DMEM/F-12, 1 mg/mL BSA, 2% B27 100 IU/mL penicillin–streptomycin, 1% N2) supplemented with 20 ng/mL bFGF to enrich for GRPs. Enriched GRPs were frozen at 2 million cells/mL in freezing medium (80% GRP basal medium +10 ng/mL bFGF +10% Chick Embryo Extract +10% DMSO) at −80 °C. Following freezing, GRPs were thawed and plated on PLL/laminin-coated culture dishes in complete medium (basal medium supplemented with 20 ng/mL bFGF) and expanded to passage 3 (P3).

### GRP cell aggregate culture and migration assay

After trypsinization, 2 million GRPs were incubated in 20 mL basal medium in a 50 mL conical tube for 1 day to allow for the spontaneous formation of aggregates. A P-100 pipette was used to gently aspirate 100 μL cell suspension with cell aggregates from the bottom of the tube, and the cell suspension was applied to the surface of PLL-coated cover glass. Cells were incubated for 1.5 hrs to allow the aggregates to attach to the cover glass. About 2 mL GRP basal medium was gently added to each dish and cultured overnight, followed by fixation with 4% paraformaldehyde. Phase contrast images were captured using a Zeiss microscope. For each cell aggregate, the diameter of the area covered by original cell sphere (d) and the area of cells that migrated out of the original sphere (D) was measured using the Zeiss software Zen.

In some experiments, cell aggregates were manually plated in order to obtain uniform cell aggregate size. Briefly, 3 million GRPs were resuspended in 20 mL GRP basal medium. After gentle mixing, 0.4 mL cell suspension was dropped onto PLL-coated cover glass, and immediately placed into the incubator to avoid evaporation of the liquid portion of the cell droplet. After two hrs, 2 mL of fresh GRP basal medium supplemented with different protein factors was added into each dish and cells were cultured overnight, followed by fixation and staining of cells with anti-nestin (Mouse monoclonal, BD Pharmigen) and DAPI.

### Stripe assay

CSPG stripes were prepared as previously described^31^. Briefly, the cover glass of a 35 mm glass bottom dish (P35G-1.5-14-C, Mattek, MA) was first coated with PLL (100 μg/mL), and printed with CSPG stripes using a PDMS gel block imprinted with micro channels (50 μm width x 3–5 μm deep). Then, 10 μL of CSPG (50 μg/mL) was added to one side of the PDMS gel block, allowing the channels to be passively filled with the liquid under capillary action, followed by air-drying overnight. This method allowed for the formation of a CSPG covered area (CSPG-on stripes) and a non-CSPG covered area (CSPG-off stripes). After washing with ddH_2_O, the cover glass was coated with BSA (50 μg/mL) overnight and rinsed with ddH_2_O. Cells were plated on the glass bottom dish with stripes at a density of about 50,000 cells/dish. Images were acquired using a Zeiss fluorescent microscope and processed in Photoshop. To better show the morphology of cells in the fluorescent and bright field overlaid image, the CSPG-off area of the fluorescent channel was cleared without affecting the CSPG-on areas.

### Spinal cord injury and transplantation of GRPs

GRPs were transplanted into a rat C4 DCH model of SCI that created a distinct cavity^14^,^15^,^20^. Briefly, the animal was anesthetized, the skin was incised (2–3 cm incision), the dorsal fat pad was retracted, and spinal musculature was reflected from the dorsal spine. A laminectomy was performed with micro-rongeurs at the C3/C4 spinal level. The dura was incised at the midline. A cavity of about 1 × 0.5 × 0.5 mm was generated using a G30 needle. GRPs from transgenic rats expressing the human placental alkaline phosphatase (AP) transgene were suspended in sterile GRP basal media/vitrogen (200,000/μL) and injected into the lesion cavity via a Hamilton syringe (2–3 μL/injection). Immediately after transplantation, the dura was closed with two 9–0 sutures. A piece of BioBrane was placed over the lesion site and the wound was closed in layers with the muscle sutured with 5–0 Vicryl and the skin stapled with wound clips. Post-surgical pain management was achieved using Buprenex before completion of surgery and twice per day for the next two days post-operatively. Wound clips were removed from the rats 3 weeks after transplantation. In those experiments where GRPs were transplanted into uninjured rats, a similar procedure was applied except that no injury was made and only 20,000–30,000 cells were injected into a similar region of the spinal cord. All rats with or without cell transplants received daily subcutaneous injection of the immune suppressor cyclosporine A (CsA, 1 mg/100 g) beginning 3 days before the surgery till end of the experiment.

### Application of Chase and bFGF *in vivo*

Immediately after grafting GRPs and suturing the dura, but prior to the application of BioBrane and closure of the musculature in layers, lentiviral vectors (~10^9 ^TU/mL) expressing bFGF, Chase, or control GFP were injected 0.5–1 mm rostral to the injury site in the dorsal spinal cord in order to generate a rostral-caudal gradient of the exogenous factor. The lentivirus vectors for GFP and Chase had been used in our previous studies^14^,^15^,^32^. They were prepared utilizing standard protocols by the UNC Viral Vector Core (http://www.med.unc.edu/genetherapy/vectorcore), and frozen at −80 °C until use. The viral vector expressing the factor was injected into the spinal cord white matter (dorsal column ipsilateral to the graft) using a sterilized Hamilton syringe attached to a pulled glass needle (0.5 μL/injection for two injections at 1 mm and 0.5 mm deep, respectively). The needle was allowed to dwell in place for 5 min after the injection and slowly withdrawn. Expression of the viral vector and the distribution of AP^+^ GRPs were analyzed by standard histology 4 weeks after transplantation.

### Tissue preparation and histological analysis

After euthanasia with an overdose of Euthasol (Virbac Animal Health, Fort Worth, TX), rats were perfused transcardially with 100 mL of ice-cold 0.9% saline, followed by 500 mL of ice-cold 4% paraformaldehyde in phosphate buffer (pH 7.4). Spinal cords were dissected and post-fixed in 4% paraformaldehyde overnight. After cryoprotection in 30% sucrose/0.1 M phosphate buffer at 4 °C for at least 3 days, spinal cord tissues were embedded in Shandon M-1 Embedding Matrix (Fischer Thermo Scientific) and cut sagittally in 20 μm sections. Tissue was collected on gelatin-coated glass slides and stored at 4 °C until use.

Slide-mounted tissue sections were stained using AP histology to visualize transplanted GRPs as described previously^13^,^14^,^20^. Slides were coversliped with Vectashield (Vector Laboratories, Burlingame, CA) and were observed with a Leica DMBR microscope equipped with a cooled CCD camera (Leica Microsystems). For immunofluorescent staining, sections were treated for 5 min in 0.2% Triton/PBS, washed three times in PBS for 5 min, and then blocked in 10% goat serum/PBS for >1 hr at room temperature, followed by overnight incubation with anti-AP (1: 400 in 2% goat serum/PBS, Chemicon) and anti-GFP (1:500 in 2% goat serum/PBS, Molecular Probes) at room temperature. After being washed three times with PBS to remove unbound antibody, sections were incubated with secondary antibodies for 2 hrs, washed three times with PBS, coated with Vectashield containing Dapi (Vector Laboratories), and overlaid with a coverslip. Slides were visualized using a Leica DM5500B fluorescent microscope (Leica Microsystems) with a Retiga-SRV camera (QImaging) and selected images were captured using Slidebook software (Olympus).

### Quantification of directional migration of grafted cells

In sagittal sections of spinal cord tissue, 6 successive zones of 500 μm in width from each side of the lesion center were analyzed (12 zones altogether). The total number of GRP cells in each zone was measured as the total intensity of AP histology staining in the zone using the software Image J after setting the proper threshold. The relative distribution of GRPs across the 12 zones was calculated based on the percentage of cells in each zone relative to total cells in the whole 12-zone region. A Directionality Index was defined as (R − C)/(R + C), where R and C are the summation of cells of 6 zones at the rostral or caudal side, respectively. In each animal, data from 3–4 sections were averaged for calculation of the relative distribution and the Directionality Index, and results from multiple independent animals from each treatment group were further averaged to represent the result of each treatment.

## Results

### CSPG inhibits the adhesion and migration of cultured GRPs

**Figure 1:**
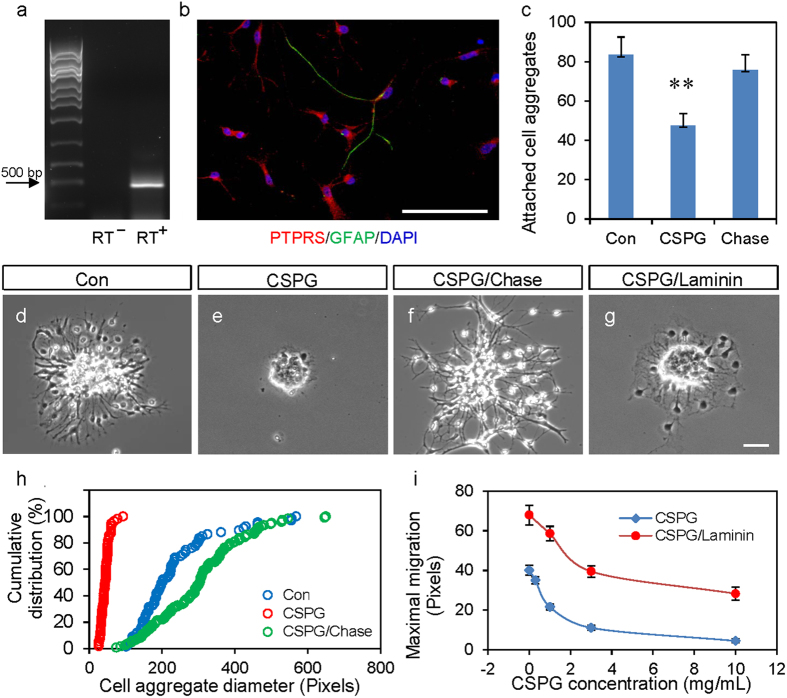
CSPG inhibits the adhesion and migration of GRPs. (**a**) RT-PCR analysis showing the expression of PTPRS in cultured GRPs using two specific primer pairs. RT^+^ indicates PCR with reverse transcription product as template. RT^−^ indicates the parallel negative control reaction without reverse transcriptase, as template. (**b**) Immunofluorescence staining of cultured GRPs with anti-PTPRS (red) and anti-GFAP (green) antibodies. Nuclei were counterstained with DAPI (blue). Note that both GFAP+ and GFAP- GRPs express PTPRS. Scale bar, 75 μm. (**c**) Quantification of the number of GRP cell aggregates on cover glass treated with different substrates. Con: PLL (poly-l-lysine, 100 μg/mL) coated cover glass; CSPG: CSPG (3 μg/mL) treatment after coating with PLL; Chase: Treatment with Chase conditioned medium after CSPG treatment. **p < 0.005 (*t*-test). (**d**–**g**) representative images showing the effect of different treatments on the migration of GRPs from the cell aggregate. GRPs were incubated in suspension for 1 day to allow the formation of cell aggregates, which were plated onto cover glass coated with CSPG or laminin (15 μg/mL). CSPG/Chase: cover glass was coated with CSPG, followed by treatment with Chase condition medium overnight before plating GRP cell aggregates; CSPG/laminin: Cover glass was coated with CSPG (3 μg/mL) and laminin (15 μg/mL) was applied into the culture medium. All cover glass were pretreated with PLL. (**h**) Cumulative distribution of the diameter of GRP aggregates after overnight culture on different substrates. (D) Dose effect of CSPG on GRP migration from cell aggregates and the mitigation of CSPG inhibition by bath application of laminin (10 μg/mL). Data are mean ± SEM.

Membrane bound CSPG has been shown to inhibit axon growth through specific receptors such as receptor tyrosine phosphatase sigma (PTPRS)^33^,^34^. RT-PCR analysis of mRNA extracted from GRP cultures detected the expression of PTPRS (Fig. 1a). We further confirmed this expression using immunofluorescent staining on cultured GRPs, both in cells positive and negative for GFAP (Fig. 1b), indicating that GRPs indeed express the CSPG receptor PTPRS.

When GRP cell aggregates were plated on cover glass coated with CSPG (3 μg/mL), significantly fewer cell aggregates were able to attach to the cover glass and grow compared to cell aggregates plated on cover glass coated with poly-l-lysine (PLL, Fig. 1c). Consistent with studies that have shown that CSPG inhibition of axon growth can be blocked by Chase treatment (see Supplementary Methods), which digests the glycosaminoglycan chains in proteoglycans^35^, Chase treatment blocked the inhibitory effect of CSPG on the attachment of GRP aggregates to the cover glass (Fig. 1c), indicating that the inhibition of GRP adhesion by CSPG is mainly mediated by the glycosaminoglycan chains. For GRP aggregates that have attached to the cover glass, the migration of GRPs out of cell aggregates was significantly suppressed by CSPG in a dose-dependent manner (Fig. 1d,e,h,i). Surprisingly, the inhibition of GRP migration was reversed by pre-treatment of the CSPG-coated cover glass with Chase. As shown in Fig 1f,h, GRPs plated on Chase-treated cover glass that had been coated with CSPG exhibited a greater migratory distance compared to PLL-coated cover glass.

The adhesion molecule laminin has been reported to modulate the axonal response to guidance factors^36^,^37^. Indeed, we found that CSPG-mediated inhibition of GRP migration was mostly mitigated by the application of the adhesion molecule laminin (10 μg/mL) directly into the culture medium 2 hrs after plating cell aggregates (Fig. 1g,i), suggesting that the specific signaling cascade activated by laminin interferes or competes with the inhibitory signal initiated by CSPG.

**Figure 2:**
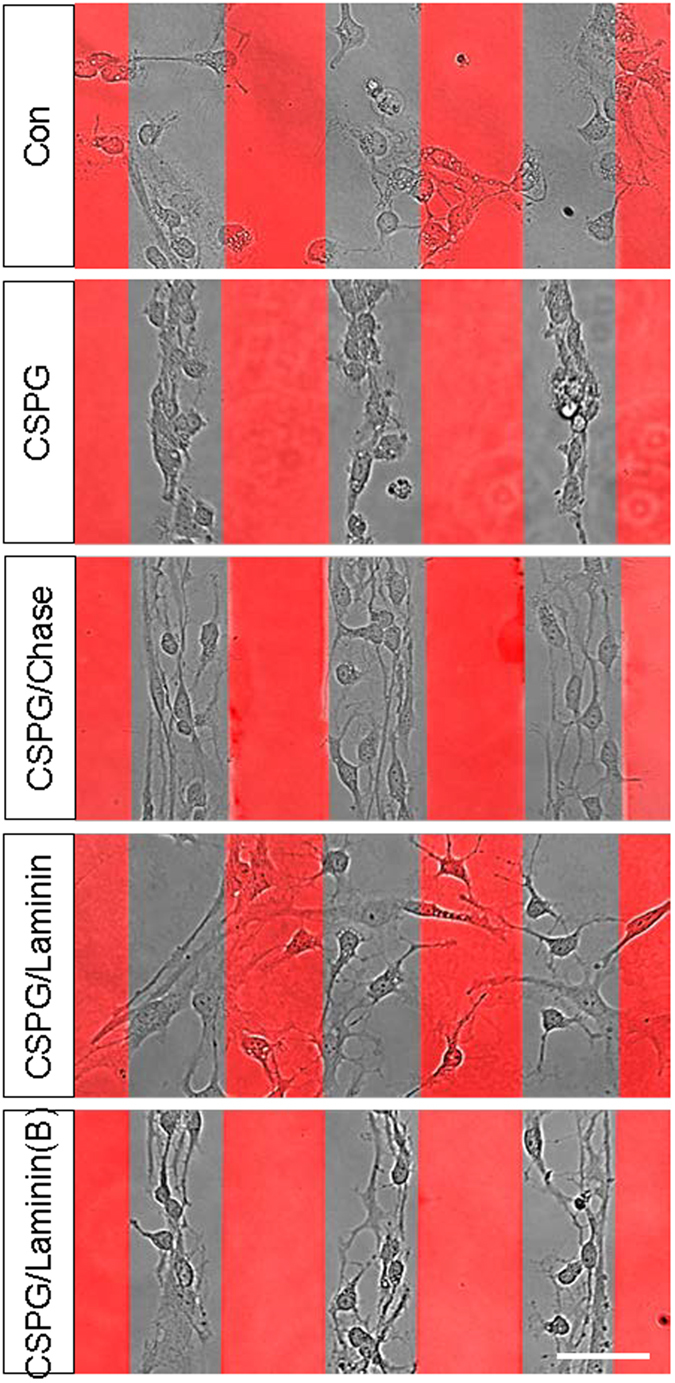
Repulsive effect of CSPG on GRP migration and the modulatory effect of Laminin on CSPG-mediated repulsion. GRPs grew along the CSPG-off stripes and avoided the CSPG-on (red) stripes. No such effect was seen when cells were plated on cover glass printed with BSA (control). Chase treatment improved lamellipodial extension of GRPs on the CSPG-off stripes, but did not block GRP aversion to CSPG-on stripes. If the cover glass with CSPG stripes was further treated with laminin (15 μg/mL), GRPs migrated extensively and ignored the inhibitory effect of the CSPG-on stripes, indicating that laminin treatment before plating the cells could “mask” the repulsive effect of CSPG. Bath application of laminin (30 μg/mL, CSPG/Laminin(B)) during culturing improved the lamellipodial extension of GRPs on the CSPG-off stripes, but did not block GRP aversion of CSPG-on stripes. Scale bar, 50 μm. Note, Laminin(B) indicates bath application of Laminin.

We further tested whether CSPG alters the migration of GRPs by monitoring the growth of cultured GRPs at the border of CSPG stripes that were printed on cover glass (see Methods), with BSA-printed stripes serving as controls. As shown in Fig. 2, GRPs grew along the CSPG-off stripes and avoided the CSPG-on stripes. These results further support the notion that CSPG is a potent repulsive factor to GRP migration. When CSPG stripes were treated with Chase (72 hr treatment at 37 °C), GRP growth was greatly improved, as reflected by markedly more elaborated lamellipodia and cell protrusions (Fig. 2). However, few cellular protrusions were able to invade the CSPG-on stripes (Fig. 2), indicating that Chase treatment was not able to completely remove the inhibitory effect of CSPG.

Similarly, bath application of laminin also improved GRP growth without blocking the repulsive effect mediated by CSPG stripes (Fig. 2, CSPG/Laminin(B)). In contrast, if the cover glass printed with CSPG stripes was coated with laminin (15 μg/mL, overnight) before plating GRPs, the CSPG-mediated repulsion on GRP migration was completely blocked, as indicated by the invasion and active extension of GRP protrusions into the CSPG-on stripes (Fig. 2, CSPG/Laminin). These effects indicate that laminin may interact with CSPG and block its inhibitory effect on GRPs.

### A screen of pro-migratory factors using the wound-healing assay

**Figure 3:**
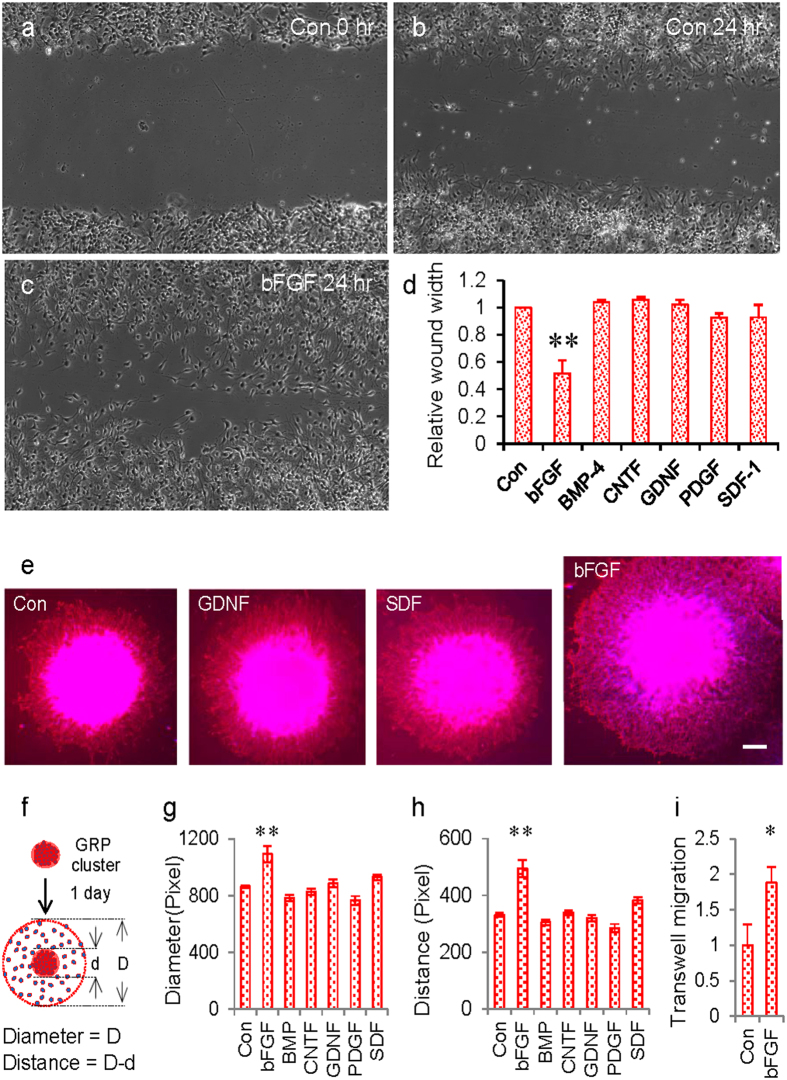
Identification of pro-migratory factors using different migration assays. (**a**–**c**) Representative images showing the wound area immediately after making the scratch, treated with or without bFGF after 24 hrs. (**d**) Summary of the “wound healing” experiment with various treatments. Different growth factors were applied to the culture medium at 20 ng/mL. bFGF was the only factor that promoted “wound healing”. (**e**) Representative images showing the effect of different factors on the migration of GRPs from cell aggregates. GRP aggregates were treated with different factors (20 ng/mL) for 24 hrs. After fixation, the culture was immunostained for the progenitor marker Nestin (red), and counterstained with DAPI (blue). (**f**) Schematic diagram showing the measurement of the diameter of the original cell aggregates (**d**) and the diameter that cells have reached (D). (**e**,**f**) The average diameter of cell clusters and maximal migratory distance under various treatments is presented. bFGF treatment significantly increased both the diameter and the maximal migration distance (D-**d**). (**i**) Normalized transwell migration of GRPs with treatment of bFGF. **p < 0.005. *p < 0.05 (*t*-test). Data are mean ± SEM.

Next, we utilized cell migration assays to screen for pro-migratory factors that promote the migration of GRPs, with special attention to factors that have been reported to guide the migration of neural/glial progenitors. As shown in Fig. 3a–d, a “wound healing” assay (see Supplementary Methods), which is used to assess cell migration, showed that treatment with basic fibroblast growth factor (bFGF, 20 ng/mL) significantly enhanced the wound healing of cultured GRPs. Other growth factors tested, including BMP4, CNTF, GDNF, PDGF, and SDF-1 (20 ng/mL for each), were not as effective as bFGF (Fig. 3d). This bFGF-mediated pro-migratory effect was also seen when GRPs were cultured as aggregates (Fig. 3g). Under this culture condition, bFGF treatment significantly enhanced the distance of GRP migration out of the aggregates (Fig. 3e–h). We further validated that bFGF can serve as an attractive factor to induce GRP migration using a standard transwell chemotaxis assay. We observed that bFGF application at the basal well (20 ng/mL) of the transwell chamber significantly enhanced the number of cells that migrated from the upper well to the other side of the filter membrane 6 hrs after the application of the factor (Fig. 3i), suggesting that bFGF can serve as a chemoattractant when it is applied in a gradient.

### Chase and bFGF improve the migration of GRPs *in vivo*

**Figure 4:**
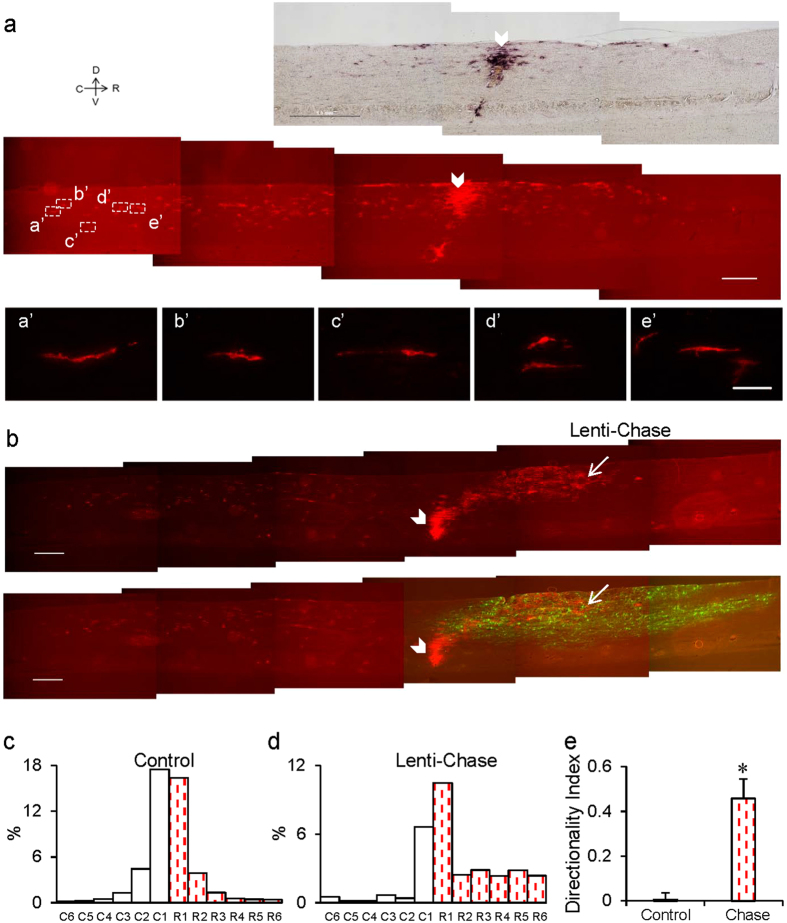
Lenti-Chase treatment induced the directional migration of GRPs grafted in uninjured spinal cord. (**a**) Survival and migration of GRPs transplanted into the spinal cord of normal adult rats. GRPs derived from embryonic AP^+^-transgenic rats were transplanted into the C4 DC of adult SD rats. The white arrowheads indicate the position of cell transplantation. AP histological staining shows the survival and migration of transplanted GRPs in both rostral (R) and caudal (C) directions along the white matter (Scale bar, 1 mm). AP immunofluorescence staining shows the morphology of transplanted cells. Some cells exhibit polarized morphology, indicating the maintenance of immature characteristics and migratory properties (Scale bar, 0.5 mm). High magnification images of selected regions (white boxes) are shown below to illustrate cells with polarized morphology (**a**’–**e**’) (Scale bar, 0.1 mm.). The farthest migratory distance of GRPs is >1.5 mm. (**b**) Induction of directional migration of GRPs in uninjured spinal cord by injection of lenti-Chase. AP^+^-transgenic GRPs were transplanted into the C4 DC of adult SD rats. Lenti-virus coding for ChaseAC-IRES-GFP was injected 1 mm rostral to the cell transplantation site. Sagittal spinal cord sections were obtained 4 weeks after the surgery. AP histo staining (left) demonstrates a rostral migratory stream of GRPs toward the Chase-GFP expressing area. The white arrow indicates the position of virus injection. Double immunostaining of AP (red) and GFP (green) shows the distribution of GRPs and Chase-GFP expressing cells. Scale bars, 0.1 mm for fluorescent images and 1 mm for the AP histology image. (**c**,**d**) Relative distribution of GRPs across 12 zones (0.5 mm wide each) from caudal to rostral of the graft site without (c, n = 5) or with (**d**, n = 2) lenti-Chase injection at the rostral side. Note the symmetric distribution of GRPs in the control group and asymmetric distribution of GRPs toward the rostral side in the lenti-Chase injection group. (**e**) Histogram showing the Directionality Index of GRP migration in normal spinal cord with or without lenti-Chase injection. Directionality Index was defined as (R − C)/(R + C), where R and C are the summation of cells of 6 zones at the rostral or caudal direction, respectively. Note that lenti-Chase injection caused directional migration of GRPs. Data in (c–e) are mean ± SEM. *p < 0.05 (*t*-test).

We next examined GRP migration after transplantation into the spinal cord of adult rats. As shown in Fig 4a,c, when AP-positive (AP^+^) GRPs were transplanted into the dorsal column (DC) of normal uninjured rat, a small population of these grafted GRPs migrated in both rostral and caudal directions. Consistent with previous findings^16^,^38^, some of these cells outside the graft site maintained a polarized morphology 4 weeks after transplantation (Fig. 4a’–e’), indicating that they were undergoing active migration. Importantly, injection of lentivirus encoding Chase (linked to a GFP cassette as the reporter gene) 1 mm rostral to the graft site induced a directional migratory stream of GRPs towards the virus injection area (Fig. 4b,d,e), indicating that GRPs are capable of guided migration into an area of diminished CSPG concentration.

**Figure 5:**
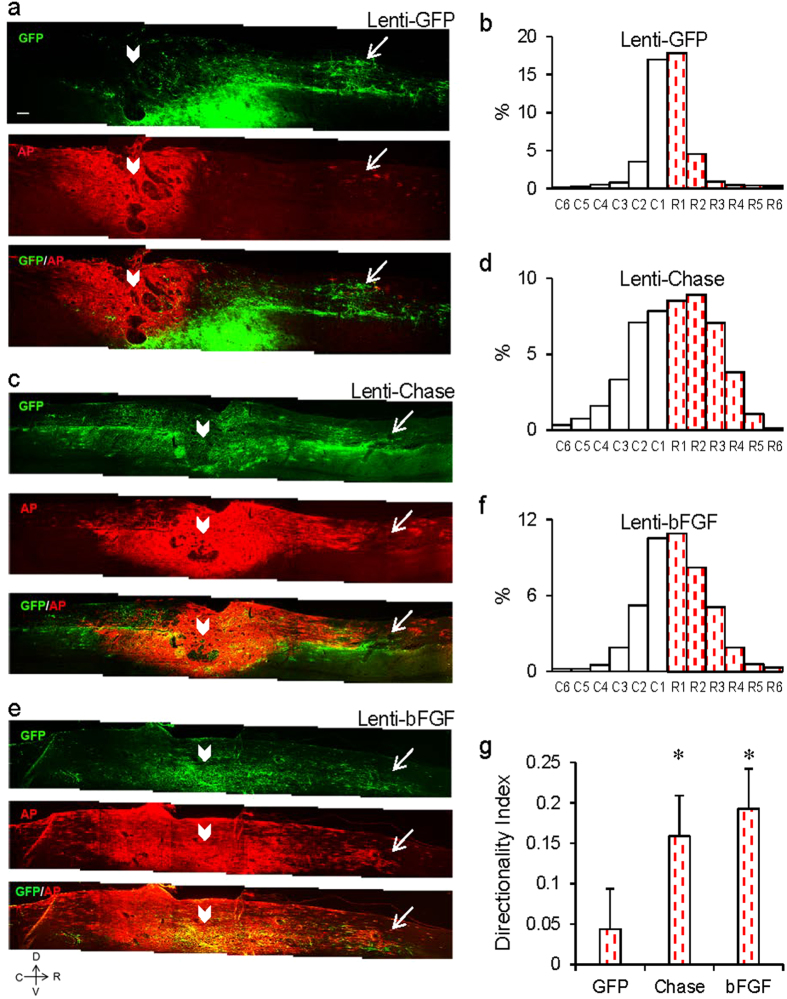
Induction of directional migration of GRPs grafted in injured spinal cord by lenti-Chase and lenti-bFGF. (**a**) Effect of lenti-GFP on the migration of GRPs transplanted into the spinal cord injury site under a DCH injury model. Virus vectors were injected 1 mm rostral to the lesion/transplant. Sagittal spinal cord sections were stained with GFP (green) and AP (red) antibodies. Most AP^+^ cells remained within the graft site and a small number of cells migrated in both rostral and caudal directions. (**c**) Effect of lenti-Chase on the migration of GRPs transplanted into the spinal cord injury site. (**e**) Effect of lenti-bFGF on the migration of GRPs transplanted into the spinal cord injury site. White arrowheads indicate GRP graft sites, and white arrows indicate virus injection sites. Section orientation: top, dorsal; right, rostral. (**b**,**d**,**f**) Relative distribution of GRPs across 12 zones from caudal to rostral of the graft with injection of lenti-GFP (**b**, n = 3), lenti-Chase (**d**, n = 3), and lenti-bFGF (**f**, n = 3) at the rostral side. Lenti-Chase and lenti-bFGF injection caused a shift of GRP distribution toward the rostral side compared with lenti-GFP, indicating induction of directional migration of GRPs. (**g**) Histogram showing the Directionality Index of GRP migration in injured spinal cord with injection of lenti-GFP, lenti-Chase, and lenti-bFGF at the rostral side. Data in (**b**,**d**,**f**,**g**) are mean ± SEM. *p < 0.05 (*t*-test) Scale bar, 100 μm.

Next, we examined the migration of GRPs following transplantation into the injury cavity in a DCH model of SCI. Immediately after the transplantation of AP^+^ GRPs into the injury cavity, lentivirus coding for different molecules was injected into the dorsal columns 0.5–1 mm rostral to the injury site. When lenti-GFP was injected, a small population of AP^+^ GRPs scattered in either the rostral or caudal direction four weeks after transplantation, with the majority of grafted cells remaining inside the lesion/transplant site (Fig. 5a,b). This distribution is consistent with previous studies in which GRPs were injected into the injury site alone, without lentivirus application^16^,^20^. When a lentivirus vector coding for Chase was injected 0.5–1 mm rostral to the injury site, a robust stream of GRPs were observed migrating toward the area expressing the reporter gene (Fig. 5c,d,g), an effect not seen in animals injected with lenti-GFP. Similar to the effect of lenti-Chase, injection of lenti-bFGF also induced a directional migratory stream of GRPs toward the areas expressing the reporter gene (Fig. 5e–g).

## Discussion

The current study shows that modulating the expression of inhibitory or attractive guidance factors around the injury/graft site is a promising strategy for inducing the directional migration of grafted cells, which may benefit axon regeneration by paving a permissive pathway composed of a migratory stream of axon-growth promoting cells. We found that CSPG inhibits the migration of GRP, which can be reversed by Chase-mediated digestion or by the presence of laminin. It is possible that such treatment could also directly reduce the CSPG-mediated inhibition of axon regeneration. We observed directional migration of grafted GRPs after locally inducing the expression of either bFGF or Chase. While transplantation of other cell types, such as OECs, MSCs, and SCs, is also a promising therapeutic modality to promote axon regeneration, it would be important to identify the attractive and repulsive factors that can effectively guide the migration of the specific cell type used for transplantation. Future studies may also consider the simultaneous manipulation of multiple factors, including attractive and inhibitory factors, in order to recruit more cells to the directional migratory stream and stimulate greater directional migration of grafted cells.

Cell migration is known to be guided by gradients of extracellular factors, either towards a high concentration of an attractive factor or away from a high concentration of a repulsive factor. The stability of the gradients of chemoattractive or chemorepulsive cues is essential to achieve an optimal guidance effect. In this study, we induced directional migration of grafted GRPs by injection of lentivirus vectors 0.5–1 mm rostral to the graft site. In uninjured spinal cord, virus vectors spread evenly in both rostral and caudal directions relative to the injection site (Fig. 4b), which may allow the formation of a gradient of the exogenous proteins where the gradient has highest concentration at the injection site. In the DCH injury model however, viral vector diffusion was not as regular as in uninjured spinal cord, and we observed broad scattering of virally-infected cells. In some injured spinal cords, we even observed infected cells caudal to the graft site. This unexpected and irregular distribution of infected cells may be attributed to the interference of the diffusion of virus vectors by injury-induced changes in the tissue environment. Under such situations, it is more difficult to form a stable gradient of guidance factors, thus explaining why only moderate levels of rostral migration were observed after injection of lenti-Chase or lenti-bFGF rostral to the lesion/transplant site in the current study. Methods of generating a more stable gradient of factors in the injured spinal cord needs to be further optimized in future studies, possibly by multiple injection sites or by better timing relative to the injury and transplantation steps.

We found that the axon repellent CSPG is also a potent inhibitory factor to GRP adhesion and migration. In the absence of additional treatment after GRP transplantation into the injury site, only a small number of GRPs could migrate out of the graft site and most grafted cells remained in middle of the injury cavity four weeks after transplantation. This lack of migration may be partly attributed to the high expression of CSPG in the adult nervous system^39^, especially within scar tissue after nerve injury^40^. Consistent with this data, we observed that lenti-Chase injection significantly enhanced the migration of GRPs from lesion/transplant site. Therefore, removing CSPG-mediated inhibition will promote the directional migration of grafted stem cells *in vivo*. Previous studies have shown that the migration of OECs transplanted into injured spinal cord was also significantly compromised compared with the extensive migration of these cells in the normal spinal cord^41^. Recent studies have also demonstrated that CSPG suppresses the migration of OECs *in vitro*^42^. It will be an important future direction to examine whether CSPG is a major inhibitory factor that restricts the *in vivo* migration of other cell types, including OECs, which are commonly used in cell transplantation therapy of SCI.

In the current study, we used Chase to remove the glycosaminoglycan chains of CSPG, a method that has been shown to eliminate the repulsive action of CSPG on axons^35^. We observed that although Chase treatment significantly improved the adhesion and migration of GRPs, it was unable to completely block the repulsive action of CSPG, as reflected by GRP avoidance of CSPG-on stripes, even after Chase treatment for as long as 72 hrs. This observation suggests that the inhibitory/repulsive effect of CSPG may be mediated by other yet unidentified functional motifs of the CSPG molecule besides the glycosaminoglycan side chain. After lenti-Chase treatment *in vivo*, this preserved repulsive action of CSPG may be responsible for the lack of migration of a large population of grafted GRPs, which stayed inside the graft site after four weeks. It is noteworthy that prior studies on axon regeneration showed a moderate pro-regenerative effect of Chase treatment^43^,^44^, which may also be attributed to the preserved CSPG inhibition that is not sensitive to Chase. Future research on axon regeneration will benefit from clarifying the structural basis of this Chase-independent inhibition by CSPG.

Laminin has been widely used as a growth-promoting substrate. Laminin-coated nanofibers have been used in peripheral nerve injury models to improve axon regeneration^45^. Laminin has also been shown to modulate the axon response to guidance factors^36^,^37^. We observed that the migration of GRPs cultured on laminin-coated cover glass is much faster than that cultured on PLL. Bath application of laminin also significantly enhanced the migration of GRPs cultured on PLL (supplementary figure S1). In our stripe assay, we observed that laminin treatment before the plating of GRPs can completely mask the inhibitory action of CSPG on GRPs, whereas bath application of laminin only mildly improved GRP migration on a CSPG substrate, unable to completely block the repulsive response of GRPs to CSPG stripes. These observations suggest that laminin can directly enhance the migration of GRPs independent of CSPG. When CSPG-coated cover glass was further coated with laminin before plating of GRPs, laminin molecules may directly bind to CSPG and mask the CSPG epitopes which elicit the repulsive action. Bath application of laminin, however, may interfere with the downstream signaling of CSPG either by competing with CSPG receptor binding or through crosstalk in convergent downstream signaling pathways, such as the RhoGTPases. It would be of great interest to explore whether application of laminin *in vivo* could also mask the inhibitory effect of CSPG. The recently developed hydrogel-releasing technique may be applied to locally release laminin into tissues surrounding the injury site^46^ and could be adapted for examining the effects of laminin on migration following injury.

Another potential strategy of suppressing CSPG inhibition is to block the receptor for CSPG or its downstream signaling cascade. Several CSPG receptor proteins have been identified, and we found that GRPs express the CSPG receptor PTPRS. An interesting future direction will be to test whether these receptor proteins actually mediate the repulsive action of CSPG on GRPs and whether transplantation of GRPs from PTPRS knockouts/knockdowns or the use of peptides that neutralize the receptor interactions^33^ will result in significantly improved migration of GRPs and a better pro-regenerative effect.

In summary, the current study has identified CSPG as an important endogenous factor that inhibits the migration of grafted GRPs beyond the SCI area, and highlights the importance of developing novel strategies to mitigate CSPG-mediated inhibition. We also established a method of inducing the directional migration of grafted GRPs in a SCI model using lentivirus-mediated expression of guidance factors, a method that can be used in combination with other therapeutic interventions to improve axon regeneration.

## Additional Information

**How to cite this article**: Yuan, X.-B. *et al.* Guiding migration of transplanted glial progenitor cells in the injured spinal cord. *Sci. Rep.* **6**, 22576; doi: 10.1038/srep22576 (2016).

## Supplementary Material

Supplementary Information
