# Supplementary material for: Guiding migration of transplanted glial progenitor cells in the injured spinal cord
Source: Sci Rep. 2016 Mar 14;6:22576. doi: 10.1038/srep22576 (PMC4789737; doi:10.1038/srep22576)
Supplement: Supplementary Information [file srep22576-s1.doc]

**Guiding migration of transplanted glial progenitor cells in the injured spinal cord**

Xiao-bing Yuan^1,2^, Ying Jin^1^, Christopher Haas^1^, Lihua Yao^1^, Kazuo Hayakawa ^1^, Yue Wang^2^, Chunlei Wang^2^, Itzhak Fischer^1^

1 Spinal Cord Research Center, Department of Neurobiology and Anatomy, Drexel University College of Medicine, Philadelphia, PA

2 Hussman Institute for Autism, Baltimore, MD

**Corresponding authors**:

Xiao-bing Yuan, PhD

Hussman Institute for Autism

Baltimore, MD

or

Itzhak Fischer, PhD

Department of Neurobiology and Anatomy

Drexel University College of Medicine

Philadelphia, PA

**Supplementary information**

**Supplementary methods**

*RT-PCR*

Total RNA was extracted from P3 GRP cultures using the Trizol reagent (Invitrogen, Carlsbad, CA) and treated with DNase I to remove potentially contaminating genomic DNA. About 300 ng of total RNA was converted to cDNA with a RevertAid First Strand cDNA Synthesis kit (Thermo Scientific Molecular Biology, ON, Canada), and 1/20 of the cDNA was used in 20-μL PCR reactions. Primers for the PTPRS were: 5'-CCCGTGCTGGCCGTGGTCTTCA-3', and 5'-CCTGCGTGGCGATGTATGCGTTCT-3'. The specificity of the PCR was validated by sequencing the PCR product.

*Wound healing assay*

About 80,000 cells were plated onto PLL-coated glass bottom dishes and cultured for 3 days in complete medium to form a monolayer. A P-10 pipette tip (Thermo Fisher) was used to make two scratches perpendicular to each other in middle of each dish. The formation of a smooth cross-shaped wound area was checked under the microscope, and only dishes with smooth wound borders were used for factor treatment. After rinsing the culture once with PBS, basal medium supplemented with 20 ng/mL of different factors (bFGF, BMP, CNTF, GDNF, PDGF, SDF; PeproTech, Rocky Hill, NJ) was applied to each dish with three parallel dishes for each treatment. After culturing for 20 hrs, the culture was fixed in 4% paraformaldehyde. Images were captured using an Olympus CK40 phase contrast microscope equipped with a CCD camera. In each dish, 3 measurements of the wound width were made in each of the four arms of the cross-shaped wound area and data were averaged to represent the wound width of each dish.

*Treatment with Chase in culture*

GRPs were cultured in basal medium and infected with lentivirus encoding the recombinant humanized chase (ChaseAC, lenti-Chase) for 2-6 days, which has been shown *in vitro* and *in vivo* to digest chondroitin sulfate proteoglycans ^1^. Conditioned medium was collected and cleared by centrifugation and stored at -20^o^C before use. To treat CSPG-coated cover glass, 0.5 mL conditioned medium was added to the surface of the cover glass and incubated at 37^o^C for 24-72 hrs. Following application, the cover glass was rinsed 3 times with PBS before GRP spheres or dissociated GRPs were plated. Conditioned medium from cultures of uninfected GRP cells served as a negative control.

*Validating Chase activity*

The effectiveness of CSPG deglycosylation by treatment with Chase-conditioned medium was performed as described previously ^1^. Briefly, nitrocellulose membranes were cut into 1 cm^2^, adsorbed with CSPG (cc117, 2.5 μg/2.5 μL; Millipore Billerica, MA), and allowed to dry at room temperature for 30 min. Membranes were incubated in blocking buffer (5% milk phosphate-buffered saline) overnight at 4°C. After two washes with 0.05% Tween Tris-buffered saline (T-TBS), the membrane was incubated for 2 hrs at 37°C with either 0.5 mL of conditioned medium from lenti-Chase-infected GRPs or an equal amount of conditioned medium from normal GRPs as control. After 3 washes with T-TBS, membranes were incubated with the 3B3 antibody (1:1000; Seikagaku Biobusiness Corporation, Japan), followed by alkaline phosphatase-conjugated goat anti-mouse IgM (1:5000; Jackson ImmunoResearch, West Grove, PA, USA) for 1 hr each. Membranes were developed for alkaline phosphatase reaction products with 100 mM Tris, 100 mM NaCl, and 50 mM MgCl2, pH 9.5, with 1.0 mg/mL nitroblue tetrazolium chloride, 0.1 mg/mL 5-bromo-4-chloro-indolyl-phosphate, and 5 mM levamisole (Sigma). The reaction was stopped by washing membranes with dH2O.

**Supplementary Figure S1. Laminin promotes the migration of GRPs**

(a-c) Representative images showing the migration of GRPs from a cell aggregate plated on cover glass coated with PLL (100 μg/mL) in the absence (a) or presence (b) of laminin (15 μg/mL) in the culture medium, or on PLL-treated cover glass which were further coated with laminin (15 μg/mL, overnight) (c). Scale bars, 100 μm. (d-e) Cumulative distribution and average value of the diameter of GRP aggregates after overnight culture on different conditions. Note that laminin treatment, either bath application or pre-coating of the cover glass, significantly enhanced the migration of GRPs. Data in (e) are mean ± SEM. **, p < 0.01 (*t*-test).


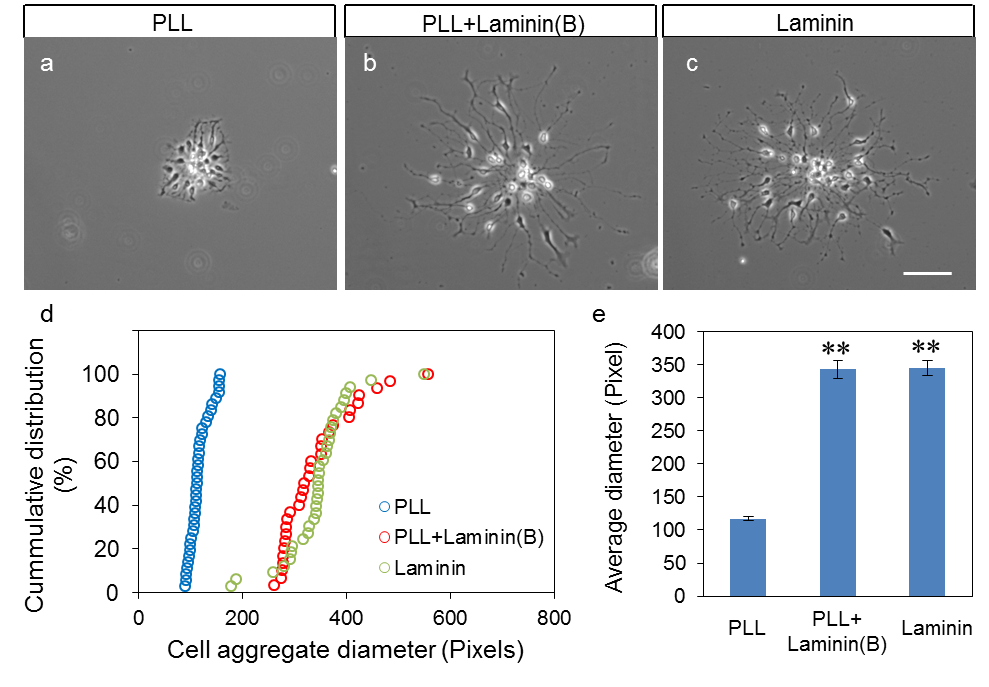


Yuan et al., Supplementary Figure S1

**References**

1 Jin, Y., Ketschek, A., Jiang, Z., Smith, G. & Fischer, I. Chondroitinase activity can be transduced by a lentiviral vector in vitro and in vivo. *J Neurosci Methods* **199**, 208-213, doi:10.1016/j.jneumeth.2011.05.007 (2011).
